# Rarity of Acute Pancreatitis as an Initial Presentation of Lung Carcinoma

**DOI:** 10.1155/cris/2913720

**Published:** 2026-01-28

**Authors:** Navin Kumar, Nayana S. Kumar, Prasoon Saxena, Ijan Dhamala, Nishit Jain, Karamveer Singh, Somprakas Basu

**Affiliations:** ^1^ Department of General Surgery, All India Institute of Medical Sciences, Rishikesh, India, aiims.edu

**Keywords:** hypercalcemia, non-small cell lung cancer, pancreatitis, paraneoplastic syndrome

## Abstract

Gallstones and alcohol consumption are the most common causes of acute pancreatitis. Lung carcinoma typically presents with respiratory symptoms, and in some cases, locoregional or distant metastases. However, acute pancreatitis as the initial manifestation of lung carcinoma is rare. Herein, we report the case of a 75 year‐old man who presented with acute pancreatitis and was diagnosed with metastatic lung carcinoma upon evaluation.

## 1. Introduction

Acute pancreatitis is common in surgical practice, and gallstones and alcohol consumption are its most common etiologies. Lung carcinoma is one of the rarest cause of acute pancreatitis, most commonly observed cases involving the small cell variant. Non‐small cell lung carcinoma (NSCLC) is an aggressive tumor with early metastatic potential and poor prognosis [1]. The probable causes of acute pancreatitis in lung carcinoma include hypercalcemia and pancreatic metastasis. Very few cases of small cell lung carcinoma have been described presenting as acute pancreatitis are reported in the literature.

## 2. Case Report

A male smoker aged 75 years presented to the emergency department with abdominal pain radiating to his back and multiple episodes of vomiting for 3 days prior to presentation. He also had a history of anorexia and weight loss of approximately 10 kg over the past 3 months. He had no history of alcohol consumption. He was afebrile with a heart rate of 88/min and blood pressure of 110/80 mmHg. Abdominal examination revealed tenderness in the epigastrium and a 3 cm × 3 cm hard nontender lymph node in the right inguinal region. The rest of the abdomen was unremarkable. The laboratory investigations revealed raised serum amylase, lipase, alkaline phosphatase, and calcium (Table 1). The rest of the blood investigations, including lipid profile, were unremarkable.

**Table 1:** Laboratory investigations.

Investigation	Value	Reference
Hemoglobin	10.2	11.0–13.0 g%
Total leucocyte count	8700	4000–11,000/cc
Platelets	110,000	150,000–450,000/cc
PT/INR	12.1/0.97	11.8/1.03
Bilirubin (total/direct)	1.1./0.26	0.3–1.2 mg/dL
ALT	79	0–35 IU
AST	88	0–35 IU
ALP	332	30–120 IU
Lipase	1790	10–140 U/L
Amylase	850	40–140 U/L
Calcium	12.5	8.4–10.3 mg/dL
Albumin	3.5	3.5–5.2 g/dL
Urea	26	17–43 mg/dL
Creatinine	1	0.55–1.02 mg/dL

Abbreviations: ALP, alkaline phosphatase; ALT, alanine transaminase; AST, aspartate transaminase; PT/INR, prothrombin time/international normalized ratio.

**Figure 1:**
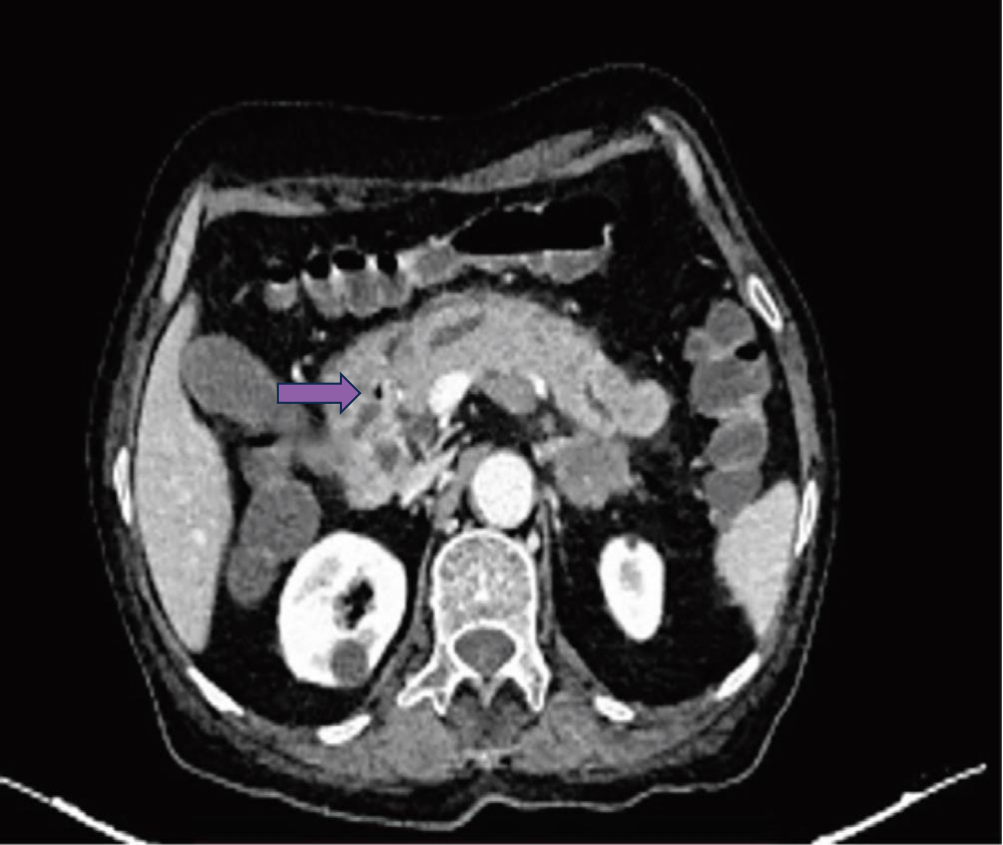
Contrast‐enhanced computed tomography of the abdomen showing acute interstitial pancreatitis (arrow) with multiple mesenteric, peripancreatic, gastro‐hepatic, and retroperitoneal lymph nodes.

**Figure 2:**
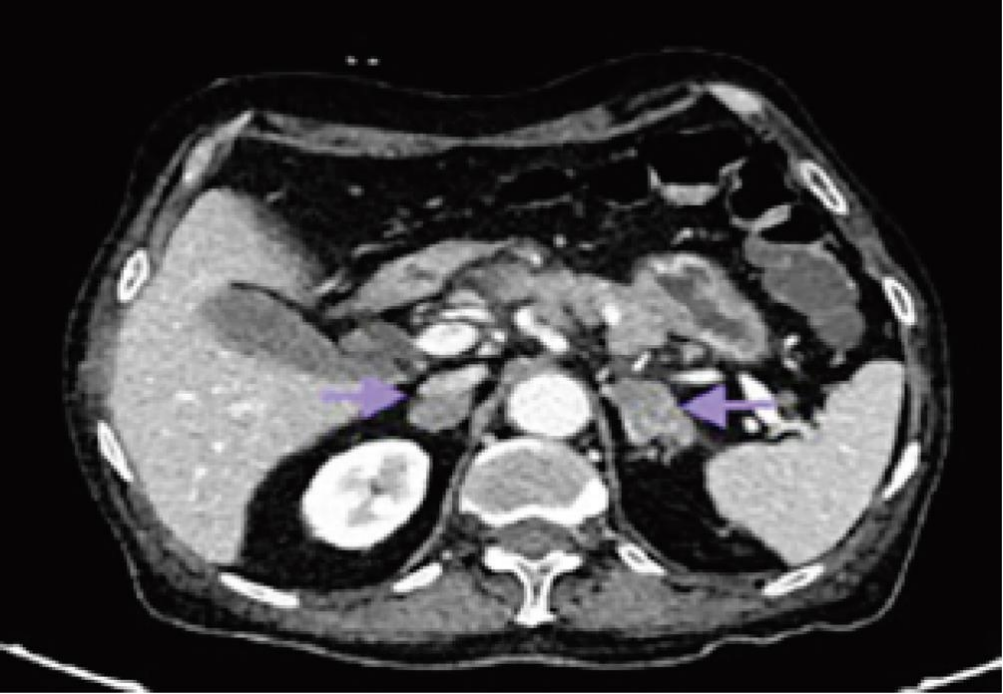
Contrast‐enhanced computed tomography of the abdomen showing metastatic bulky bilateral adrenal glands (arrows).

The Glasgow–Imrie score was two. The patient was clinically diagnosed with acute pancreatitis. Ultrasonography of abdomen revealed a single large stone in the gallbladder, and the common bile duct (CBD) was 5 mm in diameter without any stones. Hence, MRCP was not performed on the patient. Contrast‐enhanced computed tomography (CT) of the abdomen revealed Balthazar grade C acute pancreatitis with pancreatic gland abnormalities, and a peripancreatic inflammation suggesting a CT severity index of 4/10. Multiple enlarged mesenteric, peripancreatic, gastro‐hepatic, retroperitoneal, right external iliac, and right inguinal lymph nodes were noted (Figure 1). Metastatic bulky bilateral adrenal glands were also noted (Figure 2).

**Figure 3:**
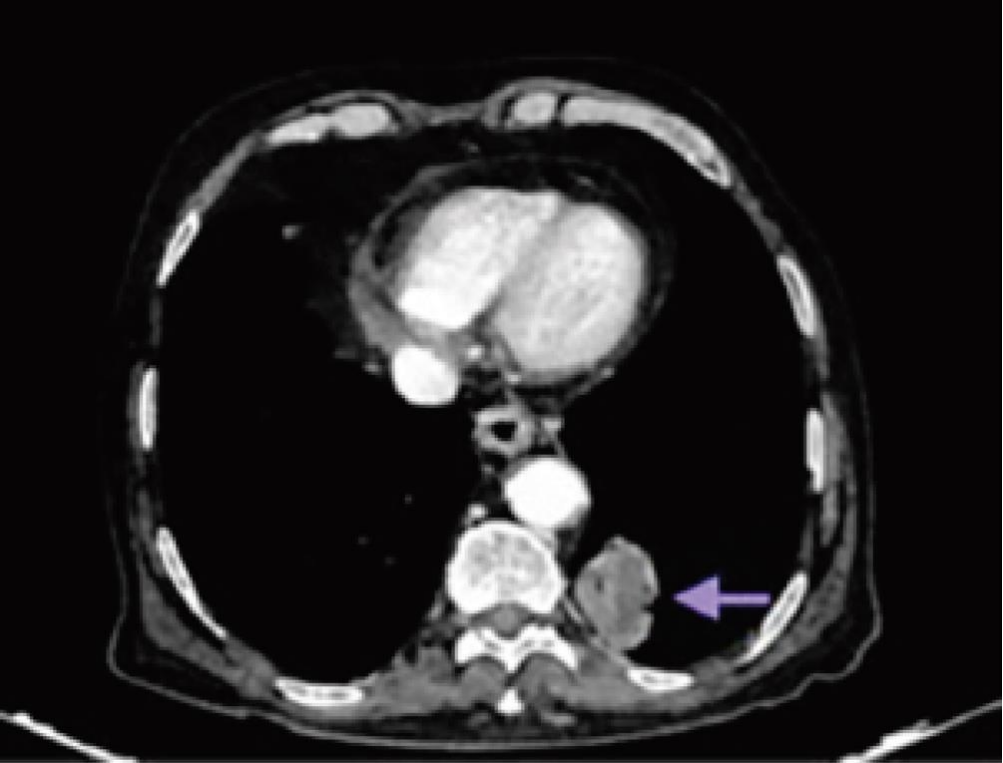
Contrast‐enhanced computed tomography of the chest showing an enhancing lesion with soft tissue attenuation (arrow) in the left lower lobe suggestive of lung cancer.

**Figure 4:**
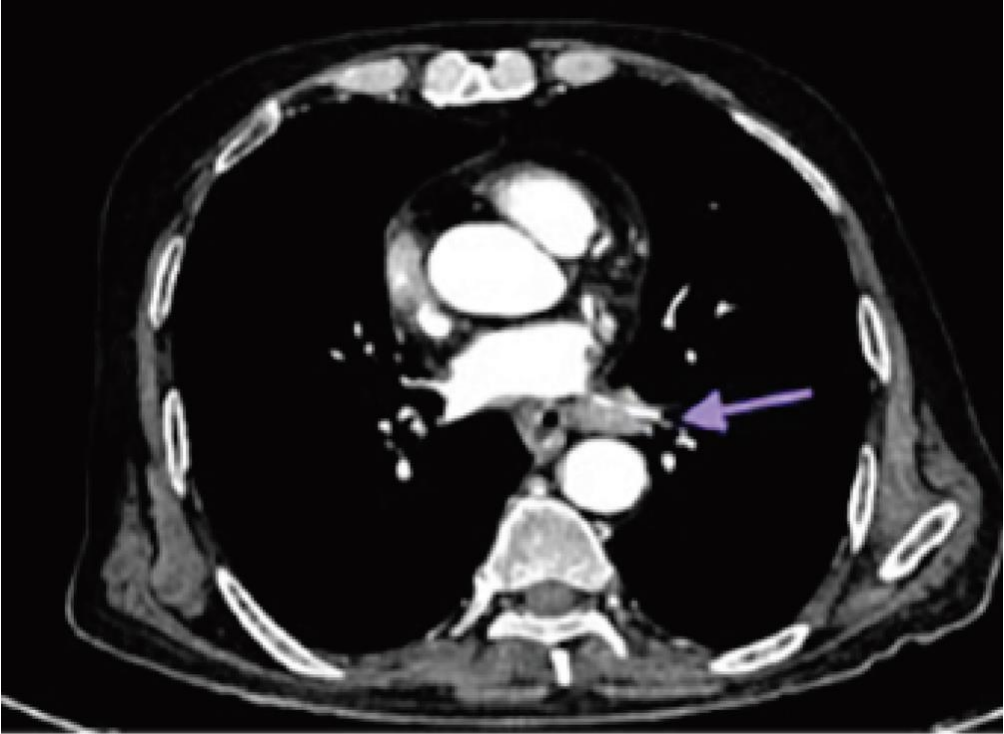
Contrast enhanced computed tomography of thorax showing multiple conglomerated, heterogeneously enhancing mediastinal lymph nodes (arrow).

CT of the chest revealed an enhancing lesion with soft tissue attenuation measuring 3.2 cm × 3.4 cm in the posterior–basal segment of the left lower lobe (Figure 3), and multiple conglomerated, heterogeneously enhancing lymph nodes in the mediastinal and bilateral hilar regions, with the largest measuring 2.8 cm × 1.8 cm (Figure 4). CECT of thorax (Figures 3 and 4) was suggestive of lung cancer.

**Figure 5:**
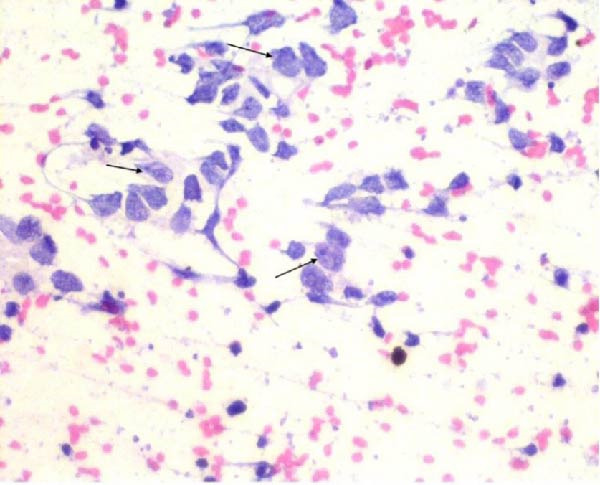
Fine‐needle aspiration cytology of the inguinal lymph node showing atypical cells with large nucleus–cytoplasm ratio, irregular nuclei, vesicular‐to‐clumped chromatin, one to two nucleoli, and scanty cytoplasm, suggestive of lymph node metastasis (arrows). Pap stain, 40x magnification.

Fine‐needle aspiration cytology of the right inguinal lymph node was suggestive of lymph node metastasis (Figure 5). Together, these findings were suggestive of stage IV metastatic lung carcinoma initially presenting as acute pancreatitis.

A histopathological diagnosis is mandatory if the clinicoradiological suspicion of malignancy is high. However, the patient’s family refused further investigation and management, and the patient was referred to palliative care.

## 3. Discussion

The two most common etiologies of acute pancreatitis are gallstones and alcohol consumption; however, hypercalcemia is a rare cause of acute pancreatitis. Acute pancreatitis is usually associated with low serum calcium levels; therefore, hypercalcemia may arise due to hyperparathyroidism or malignancy. Hypercalcemia causes pancreatic injury via secretory blockage, accumulation of secretory proteins, or activation of proteases. The cause of hypercalcemia should be accordingly managed along with the administration of intravenous fluids, ideal fluid of choice is 0.9% normal saline, initial 1–2 L of bolus followed by a maintenance rate of 200–500 mL/h according to patient’s cardiac and renal status, loop diuretics ideally furosemide (40–80 mg every 6–12 h), and bisphosphonates preferably zoledronic acid 4 mg IV over 15 min and routine management of pancreatitis and its complications. One possible cause of pancreatitis in our patient was hypercalcemia, which may be associated with a paraneoplastic syndrome of lung malignancy or metastasis [2–11]. NSCLC and small cell lung cancer account for 85%–90% and 10%–15% of lung cancers, respectively [12]. The most common extrapulmonary sites of distant metastases of lung cancer are the brain, bones, liver, and adrenal glands [13]. Approximately 20%–50% of patients with NSCLC present with metastatic disease. The prognosis of stage IV NSCLC is poor, with a median overall survival time of 7–11 months [14]. Previous autopsy studies have reported a 1.6%–5.9% incidence of metastasis to the pancreas from lung carcinoma [15]. Metastasis‐induced pancreatitis is caused by the obstruction of the pancreatic duct by metastatic deposits or pancreatic compression by the enlarged regional lymph nodes, which can cause autolysis via pancreatic enzyme activation. Vascular compromise due to the invasion of metastatic neoplastic cells is another suggested mechanism for acute pancreatitis [16]. Some case reports have described hypercalcemia as a causative mechanism for acute pancreatitis in patients with NSCLC [17–19]. On the contrary, in some cases of small cell lung cancer with acute pancreatitis, where no common causes of pancreatitis were identified, autopsy showing no evidence of pancreatic metastasis suggested paraneoplastic syndrome as a possible cause [20]. In our case, contrast‐enhanced CT of the abdomen revealed no pancreatic metastases. Gallstone‐induced pancreatitis was also a differential diagnosis because the patient had cholelithiasis. However, there was only one large stone in the gallbladder, the CBD diameter was within normal limits, there were no stones in the CBD or main pancreatic duct, and hypertriglyceridemia was ruled out. Therefore, paraneoplastic syndrome due to lung malignancy with hypercalcemia was thought to be the cause of the acute pancreatitis, which was the first and only manifestation of an underlying lung carcinoma. This is an infrequent presentation of lung carcinoma. There are very few case reports in the literature describing lung cancer presenting as acute pancreatitis due to hypercalcemia; among these, hypercalcemia was the initial and sole manifestation of lung cancer, with an often‐unknown underlying cause. Such presentations, typically, occur in the advanced stages of the malignancy [17–19]. Possible reasons for paraneoplastic hypercalcemia in lung carcinoma are the secretion of parathyroid hormone‐related protein [PTHrP] or osteolytic activity due to cytokine production, which stimulates osteoclasts and has a poor survival outcome [20, 21]. Chemotherapy is the first‐line of treatment for patients with stage IV NSCLC [14]. The NCCN Clinical Practice Guidelines recommend immunotherapy for locally advanced and metastatic lung cancer. Patients with stage III NSCLC treated with durvalumab (anti‐PD‐L1 monoclonal antibody) for upto 12 months did not progress after definitive chemoradiation therapy. Patients with NSCLC progressing with, or after, first‐line chemotherapy can be switched to nivolumab immunotherapy [22].

## 4. Conclusion

Paraneoplastic hypercalcemia due to carcinoma of the lung can present with acute pancreatitis and is usually present in the advanced stages of malignancy with poor outcomes. In the absence of common causes of pancreatitis, hypercalcemia should be appropriately investigated. Early diagnosis and management can increase the survival rates.

## Funding

This study did not receive any funding.

## Ethics Statement

This study was compliant to ethical requirement. Ethics clearance taken from the Institutional ethics committee with letter number AIIMS/IEC/24/127 dated March 15, 2024, from the All India Institute of Medical Sciences, Rishikesh.

## Consent

Written informed consent was obtained from the patient for publication of this case report and any accompanying images. Identifying details have been anonymized. Patient consented to this publication.

## Conflicts of Interest

The authors declare no conflicts of interest.

## Data Availability

The authors have nothing to report.
